# Combined Effect of Dietary Protein, Ractopamine, and Immunocastration on Boar Taint Compounds, and Using Testicle Parameters as an Indicator of Success †

**DOI:** 10.3390/foods9111665

**Published:** 2020-11-14

**Authors:** Tersia Needham, Rob M. Gous, Helet Lambrechts, Elsje Pieterse, Louwrens C. Hoffman

**Affiliations:** 1 Department of Animal Science and Food Processing, Faculty of Tropical AgriSciences, Czech University of Life Sciences Prague, Kamýcká 129, 16500 Prague-Suchdol, Czech Republic; 2 Department of Animal Sciences, University of Stellenbosch, Private Bag X1, Matieland, Stellenbosch 7602, South Africa; helet@sun.ac.za (H.L.); elsjep@sun.ac.za (E.P.); louwrens.hoffman@uq.edu.au (L.C.H.); 3 School of Agricultural, Earth and Environmental Sciences, University of KwaZulu-Natal, Scottsville 3209, South Africa; Gous@ukzn.ac.za; 4 Center for Nutrition and Food Sciences, Queensland Alliance for Agriculture and Food Innovation (QAAFI), The University of Queensland, Health and Food Sciences Precinct, 39 Kessels Rd, Coopers Plains 4108, Australia

**Keywords:** androstenone, dietary protein, GnRH, Improvac, ractopamine hydrochloride, skatole, testicles

## Abstract

This study investigates the combined effect of immunocastration, dietary protein level (low, medium or high) and ractopamine hydrochloride supplementation (0 or 10 mg/kg) on the adipose concentrations of androstenone, skatole and indole in pigs, and explores whether body mass, carcass fatness or testicular parameters may be indicators of boar taint in these carcasses. Immunocastration was successful in decreasing testicle functioning, and adipose androstenone and skatole concentrations, in all individuals. Immunocastration decreased testicle weight and length, seminiferous tubule circumference and epithelium thickness. Testicle tissue from immunocastrates was also paler, and less red in color, in comparison to non-castrated controls. Dietary protein level and ractopamine hydrochloride supplementation had no influence on the adipose concentration of androstenone, skatole and indole. Testicle size and color were moderate to strong indicators of androstenone and skatole concentrations in the carcasses, and thus vaccination success. Immunocastration together with the adjustment of dietary protein and ractopamine hydrochloride supplementation, is successful in preventing boar taint while maintaining growth performance.

## 1. Introduction

The use of surgical castration in male piglets is currently under ethical scrutiny, motivating the investigation into alternative methods to control boar taint in pork products. Boar taint is described as an unpleasant aroma and flavor in pork, and is the result of an increased production of androstenone (5α-androst-16-en-3-one) [1], skatole (3-methylindole) [2] and to a lesser extent indole [3,4] by the boar as it reaches sexual maturity. The lipophilic pheromone androstenone (5α-androst-16-en-3-one) is produced in the testicles of boars, and has the primary function of stimulating the standing reflex in sows, but it also accumulates in adipose tissue [3]. Thus, castration is used to prevent the production and accumulation of androstenone in male pig carcasses. Although a successful means of preventing boar taint, physical/surgical castration is linked to certain welfare issues, such as acute and chronic pain, wound infection, morbidity and mortalities [5]. Castration also inhibits the anabolic effect of male androgens, resulting in less efficient growth and fatter carcasses. When castration is not considered to be a viable option, entire (intact/non-castrated) pubertal males are slaughtered before attaining sexual maturity, to minimize the occurrence of boar taint in their carcasses. However, this approach results in small carcasses with narrow profit margins for producers, and in fact, does not appear to be commercially successful in decreasing the incidences of boar tainted-carcasses [6]. The production of young, non-castrated male pigs with lean carcasses poses further issues in countries where subcutaneous fat deposition is important for the production of high-quality, traditional dry-cured products [7].

When heated, the odor of androstenone is described as “ruinous”, “sweaty” and “sexual” [8], and as “fecal”, “boar”, “urine” and “perspiration” [9]. Consumer sensitivity to androstenone depends on its concentration [10], as well as the consumer’s genotype [11], ethnic group [12], and sex [8,13]. While the sensory detection threshold for androstenone is approximately 0.4 to 0.5 µg/g [14], consumer sensitivity is highly variable, from consumers being anosmic, to those being highly sensitive to androstenone at low concentrations. Regular exposure to androstenone can also induce the ability to perceive androstenone in those consumers considered to be anosmic, which in turn can further reduce consumer acceptability of pork containing boar taint [15]. Additionally, when both skatole and androstenone are present in pork, the risk of the consumer detecting boar taint and rejecting the product increases further [10]. Factors affecting skatole accumulation in adipose tissue are still largely undescribed, but it is known that skatole is produced by bacterial breakdown of tryptophan in the large intestine [1]. This microbial degradation may be affected by the digestibility of feed, as well as the extent of intestinal cell debris production, and thus skatole is not exclusively produced in male pigs [16]. However, skatole is catabolized by the liver, the enzymatic functionality of which is influenced by steroids produced by the testicles [17]. Skatole in pork produces a fecal odor [2] and typically, consumers’ odor detection threshold concentration for skatole is much lower than androstenone, and while the commonly used skatole threshold is 0.2 µg/g fat, consumers can be sensitive even at as low as 0.026 µg/g [14]. Compared to androstenone, the majority of consumers are able to perceive skatole in pork, and dislike it [8]. Currently, a number of techniques for identification of carcasses with boar taint in commercial abattoirs exist, with their own cost-implications [18].

Various approaches have been investigated to control boar taint-related compounds [19], with gene-editing showing huge potential, but also a rather lengthy timeline before commercial implementation. While some approaches may address decreasing the incidences of boar taint, they do not necessarily address the welfare issues of entire male production, such as aggressive behavior [20], the meat/product quality issues associated with entire males [21], or the application of these technologies, such as gene editing, is currently limited in various countries. Thus, commercial interest in the use of immunocastration has increased, which entails vaccinating the animal against its own gonadotropin-releasing hormone (GnRH). The production of GnRH-antibodies blocks the functioning of the hypothalamic-pituitary-gonadal axis, which in turn inhibits the production of the gonadotropins luteinizing hormone (LH) and follicle-stimulating hormone (FSH). Both of these gonadotropins stimulate steroidogenesis in the testicles, and thus immunocastration results in the inhibition of testicular androstenone production. Together with the decrease in testicular steroid production, the growth, nutrient requirements and fat deposition of immunocastrated pigs are influenced [22]. However, feeding a higher protein diet, together with ractopamine hydrochloride (β-adrenoreceptor agonist), improves feed efficiency of immunocastrated pigs, offsetting fat deposition and slowing growth rates [22]. Seeing as diet has an influence on skatole production, and the fact that most consumers are capable of perceiving this compound, the potential effect of dietary changes and feed additives on boar taint levels cannot be ignored. Thus, the combined effects of such *ante-mortem* strategies for preventing boar taint while maintaining high levels of growth productivity should be investigated, prior to their application in commercial settings. In addition to this, correlations between changes in body mass, carcass fatness, and testicular functioning in relation to adipose androstenone and skatole concentrations should be investigated. By examining these correlations, simple slaughter-line methodology for identifying carcasses with a higher predisposition for boar taint may be developed.

Thus, the aims of this study were to determine the combined effect of immunocastration, dietary protein level and ractopamine hydrochloride supplementation on adipose concentrations of androstenone, skatole and indole, as well as to establish whether body mass, carcass fatness or testicular parameters may be possible indicators of boar taint in these carcasses. 

## 2. Materials and Methods 

### 2.1. Animals, Housing and Feeding

Ethical approval for the study was obtained from the Research Ethics Committee: Animal Care and Use of Stellenbosch University (SU-ACUM13-00022). A growth performance trial was conducted with 120 entire PIC^®^ male pigs (PIC© Large White × Landrace × White Duroc maternal line bred with a PIC© 410 terminal sire), at the Elsenburg boar testing facilities (Stellenbosch, South Africa). Each individual pen consisted of a concrete sleeping area with pine wood shavings, and a separate dunging area (free from shavings) with a nipple drinker. The pens were cleaned daily, and food and water were provided *ad libitum*. Each pig was allocated randomly to one of 12 treatment combinations (10 pigs per treatment), according to a 2 × 2 × 3 factorial design. The main effects included castration status (immunocastrated (IC) versus non-castrated/entire (E) males), ractopamine hydrochloride (RAC; Paylean, Elanco^TM^ Animal Health, Pretoria, South Africa) supplementation (0 or 10 mg/kg), and the dietary protein inclusion level (low, medium and high; Table 1 and Table 2). The medium protein diet was established by mixing the low and high protein diets at a ratio of 50:50. 

The sixty pigs allocated to the IC treatment group, received 2 mL Improvac^®^ (Zoetis™ Animal Health, Sandton, South Africa) at 16 weeks of age, and again at 20 weeks. Up until 20 weeks of age, all pigs received a commercial grower diet, after which they were fed one of three experimental balanced protein diets (7.50, 9.79 and 12.07 g digestible lysine/kg; Table 1) with RAC supplementation at 0 or 10 mg/kg, for the last 28 days of growth. 

### 2.2. Slaughtering and Testicle Measurements

All pigs were slaughtered at 24 weeks of age, i.e., four weeks after the administration of the second Improvac^®^ injection. The pigs were transported to a commercial abattoir and slaughtered according to standard practices, which involved electrical stunning followed by exsanguination. According to the live weight at slaughter, 96 pigs were sampled by selecting eight pigs from the midweight-range in each treatment. The backfat depths of the 96 pigs were determined between the second and third last rib (counted from the cranial end) and 45 mm from the spine (P2 position), using a Hennessy Grading Probe (Hennessy Grading Systems, Auckland, New Zealand). A summary of the initial live weight at the start of the trial (16 weeks old), the slaughter weight at 24 weeks of age and backfat depth at slaughter for the 96 selected pigs only can be found in Table 3. Further details regarding the growth performance, carcass traits and meat quality of the pigs may be found in Needham et al. [22,23,24]. 

Their testicles were collected on the slaughter-line prior to evisceration, placed in marked plastic bags, and transported on ice to the laboratory for further processing. Upon arrival at the laboratory, the epididymis and connective tissue were removed, and each individual testicle was weighed on a RADWAG PS750/C/2 scale (Wagi Elektroniczne, Radwag, Radom, Poland; accurate to 0.001 g). Testicle measurements (length and width) were taken using a calibrated engineering caliper (150 mm Electronic Digital Vernier Caliper CE ROHS), and testicle volume was determined by water displacement [25]. 

For histological evaluation, 32 pairs of testicles were sub-sampled by selecting eight of the mid-weight animals from the following treatment combinations, fed only the medium protein diet: E fed RAC, E fed no RAC, IC fed RAC, and IC fed no RAC. Each testicle was cut alongside the widest axis, and the surface color was measured, without bloom time [25], according to the International Commission on Illumination (CIE) Lab color system. A Color-guide 45/0 colorimeter (Catalogue number 6801, BYK-Gardner GmbH, Geretsried, Germany) was used, with an aperture diameter size of 11mm and an illuminant/observer angle of 65/10°. Calibration of the colorimeter was done prior to measurement, using the black calibration standard, white calibration standard, green checking reference, and high gloss standard. The hue angle and chroma value were calculated as follows:

$$Hue-angle\;\left({}^{\circ}\right)=\;tan^{-1}\;\left(\;\frac{b^{*}}{a^{*\;}}\;\right);\;Chroma\;\left(C*\right)={\left(a^{*\;2}+\;b^{*\;2}\right)}^{-0.5}$$

Thereafter, testicle tissue samples (approximately 1 cm × 1 cm × 1 cm) were taken, preserved in 10% neutral buffered formalin, and stored for later histological processing. Slides were prepared from the preserved tissue, and stained using haematoxylin and eosin. Histology slides were evaluated at 40× magnification, using an Olympus IX70 microscope (Olympus Corporation, Tokyo, Japan). One hundred seminiferous tubules were measured per sample, and their circumference and epithelium thickness were determined using the Olympus Image Analysis Software package (Olympus Corporation, Tokyo, Japan).

### 2.3. Chemical Analyses of Androstenone (5α-Androst-16-en-3-One), Skatole (3-Methylindole) and Indole

At approximately 24 h *post-mortem*, subcutaneous backfat samples were taken from the same 96 selected pigs by removing a 2 cm thick strip of fat from the loin, at the position of the third-last rib. Samples were frozen at −20 °C, until simultaneous analysis for androstenone (5α-androst-16-en-3-one), skatole (3-methylindole) and indole following an adapted methodology [26]. At the time of analysis, the adipose tissue samples were thawed, and 5 g of each sample was cut into thin flakes before placing them into stomacher bags. An internal standard was prepared by adding 2-methylindole to methanol at a concentration of 200 µg/kg, and 5 mL of this was added to each stomacher bag. The samples were then homogenized for two minutes, using a stomacher BagMixer^®^ 400 W (Interscience, Saint-Nom-la-Bretèche, France). The supernatant was transferred from the stomacher bag into a sterile Cellstar^®^ tube (Greiner Bio-One, Kremsmünster, Austria), and cooled by submersing the tube in liquid nitrogen for approximately one minute. Subsequently, the samples were centrifuged for six minutes at 5000 rpm. Following removal from the centrifuge, the samples were frozen within their tubes using liquid nitrogen, to clear the upper phase, and then filtered using a 0.22 µm syringe filter. After this filtration, 300 µL of the extract was placed into vials for analysis, and diluted with 200 µL of 1% acetic acid. 

The samples were analyzed for 5α-androst-16-en-3-one using tandem mass spectrometry (Waters Xevo TQ triple quadruple mass spectrometer, Waters Corporation, Milford, CT, USA), and for skatole and indole using ultra-performance liquid chromatography with fluorescence (Waters ACQUITY UPLC FLR Detector, Waters Corporation, Milford, CT, USA). A Kinetex C18 column was used (2.6 um, 150 × 2.1 mm, Phenomenex Inc., Torrance, CA, USA) with two solvent gradients: 7.5% formic acid and 49:49:2 methanol:acetonitrile:isopropanol. Sample injection volume was 10 µL, and the column temperature was set to 40 °C. Standard curves were established for each of the compounds analyzed, such that the calibration range and limit of quantification for 5α-androst-16-en-3-one was 0.01 to 13 µg/g and 0.02 µg/g, respectively, whereas the calibration range and limit of detection for both skatole and indole was 0.008 to 0.08 µg/g and 0.004 µg/g, respectively.

### 2.4. Statistical Analyses

Data were analyzed using STATISTICA (Version 13.5.0, StatSoft Inc., Tulsa, OK, USA), together with integrated R software (R Foundation, Vienna, Austria). Normality of the data was evaluated, and those which were not normally distributed (testes volume, indole and skatole concentrations) underwent Box-Cox transformation. One-way analysis of variances (ANOVAs) were established for each parameter using the R “lm” function, with the treatments (castration status, dietary protein level and ractopamine supplementation) as the fixed effects, and the animal as the random effect. In the case of testicles color and histology data, only castration status was included as a fixed effect. Treatment means were compared using Fishers LSD post-hoc testing. Correlations between all parameters were established in Statistica, using Spearman’s Correlation Coefficients, and visually displayed using a correlation heatmap with cluster dendrograms, generated with R software (function: “heatmap.2”). A significance level of 5% was used throughout. Further descriptive statistics were performed for the average concentration range and percentage of samples above the analytical detection limit for androstenone, skatole and indole. 

## 3. Results

**Figure 1:**
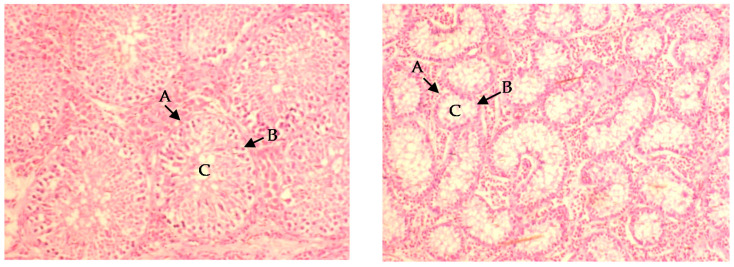
Micrographs of testicular tissue for entire (**left**) and immunocastrated (**right**) pigs slaughtered at 24 weeks of age, 4 weeks after the second immunocastration vaccination with Improvac (40× magnification). Immunocastration resulted in atrophy of the seminiferous tubules (A), loss of spermatocytes (B), and increased lumen size (C).

Immunocastration decreased the testicles’ weight (*p* < 0.01) and length (*p* < 0.01), but did not influence testicles’ volume and width (Table 4). Dietary protein level and RAC had no influence on testicle size. Furthermore, immunocastration decreased seminiferous tubule circumference (*p* < 0.001) and epithelium thickness (*p* = 0.002), resulting in increased lumen size and deformation of the seminiferous tubules (Figure 1). The results for the CIE Lab color values indicated that the testicles from immunocastrates had higher L* values (*p* < 0.001), higher b* values (*p* < 0.001), and lower a* values (*p* < 0.001; Table 4). Thus, they were paler, more yellow and less red when compared to the surface color of testicles from entire male pigs. Supplementation with RAC had no influence on testicles color and histology.

Immunocastration decreased the subcutaneous backfat concentrations of androstenone (*p* < 0.01) and skatole (*p* < 0.01), but not indole (Table 5). For both the immunocastrated and entire male carcasses, no adipose tissue had androstenone concentrations over the sensory threshold (0.426 µg/g fat) [14]. However, 48% of entire males had mean adipose skatole concentrations above the sensory threshold (0.026 µg/g fat) [14], while the average immunocastrated adipose tissue skatole concentrations were below that of the sensory threshold, with only two animals exceeding the sensory threshold with concentrations of 0.034 and 0.028 µg/g fat. There was no effect of RAC supplementation or dietary balanced protein level on the levels of skatole.

**Figure 2:**
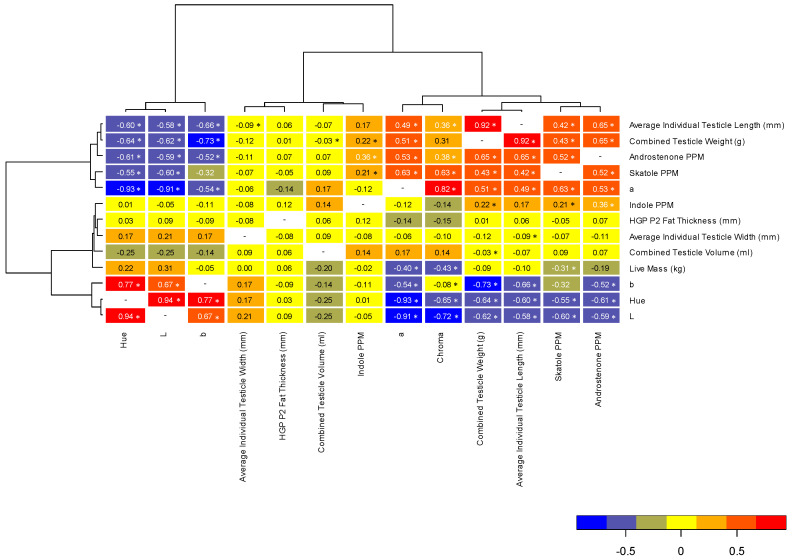
Pearson’s correlation coefficient heatmap for body mass, Hennessy Grading Probe backfat thickness (HPG at P2 location), testicle size and color (L*, a*, b*, hue and chroma) parameters and backfat concentrations of androstenone, skatole and indole. Linkage distance within the clustering analysis indicates how closely the correlations of these variables match one another. The legend indicates the strength and direction of the correlations between the parameters, and Spearman’s correlation coefficients are shown between parameters. Significant differences are indicated by asterisks, at *p* < 0.05.

Correlations between all parameters are represented in Figure 2, with the variables showing three primary groupings. The first grouping (average testicle length, combined testicle weight, androstenone concentration, skatole concentration and a* color values), showed moderate to strong positive correlations with each other, as well as chroma values. The second grouping (indole concentration, P2 backfat thickness, average testicle width, testicle volume and live mass) showed no, or weak, correlations with all other variables. The third grouping (b* color values, hue angle and L* color values) showed moderate to strong negative correlations with the first group of variables (average testicle length, combined testicle weight, androstenone concentration, skatole concentration and a* color values). The linkage distance of the clustering analysis indicates how closely the correlations of these variables match one another. For example, average testicle length and combined testicles weight showed the most similar correlations to one another. Regarding potential indicators of androstenone, skatole and indole concentrations, testicle parameters (weight, length and color values) were moderately to strongly correlated; however, live mass and carcass fatness show weak correlations with boar taint compound levels (Figure 2).

## 4. Discussion

The immunocastration vaccination schedule used was successful in decreasing testicular functioning, as indicated by the decrease in weight, disruption of the seminiferous tubule morphology, color changes and decreased androstenone production. Previous results have shown seminiferous tubule atrophy and spermatocyte loss in immunocastrated pigs [27], as well as sheep [28,29,30], and cattle [31,32]. Immunocastration also caused the testicle tissue surface color to become paler, less red and more yellow, as similarly reported [25]. These changes in testicle tissue color are likely indicative of their decreased functioning after immunocastration and, together with testicle weights, may provide an indication of vaccination success [25] and subsequently risk of boar taint in the carcass. Immunocastration decreased adipose androstenone concentrations, to values comparable with previous studies using similar vaccination schedules [25,33]. Immunocastration also decreased adipose skatole levels, which is expected, as androstenone inhibits the skatole-induced expression of the CYP2E1 enzyme involved in skatole metabolism [34]. However, diet and supplementation with ractopamine hydrochloride had no influence on skatole levels in the present study, which was somewhat unexpected, as changes in dietary protein and fiber contents may influence the gut pH and microflora [35] and intestinal cell debris production [16], all of which are thought to contribute to the amount of skatole production in the gut. Furthermore, the use of feed additives may also potentially have an influence on the enterohepatic recirculation of androstenone as well as on gut microflora, influencing skatole production [19]. However, skatole synthesis and metabolism is a complex process of which the influence of various factors on this remain poorly understood [36], and it is likely that the dietary factors included in the present study were not detrimental to the gut pH, microflora and metabolism of boar taint-related compounds. 

The adipose androstenone concentrations in the carcasses of entire males within the present study were also low, despite their slaughter weight (~125 kg), compared to the concentrations reported in the control males of other immunocastration studies [25,33]. Whilst variation in these reported values are expected, considering the different analytical methodologies used, it is likely that genotype influenced the androstenone levels reported in the present study compared to other studies. For example, Weiler et al. [33] used progeny from a Large White x Landrace maternal line and Pietrain terminal sire line in their study, and reported an average fat androstenone level of 1.75 µg/g in entire males. Whereas Lealiifano et al. [25] used Large White × Landrace boars, and reported an average fat androstenone level of 0.91 µg/g. A recent study also describes differences in adipose androstenone levels of slaughter pigs from three different sire lines at slaughter (after immunocastration), indicating that differences in growth rate, maturity at slaughter weight and lean growth potential of each genotype influences the adipose androstenone levels [37]. It is also accepted that heavier pigs have a higher risk of elevated androstenone levels, as described by Pieterse [38], who found that when slaughter weight was increased from between 102–113 kg to 133 kg over five different genotypes of boars, adipose androstenone levels increased concomitantly. Despite differences in genotype and weights between studies, it is also possible that in the absence of female pigs in the housing system in the present study, the boars were not stimulated to produce testicular steroids and thus androstenone. Nonetheless, despite the low levels of androstenone in entire males in the present study, immunocastration successfully decreased the adipose skatole levels compared to entire males, the latter having nearly 50% of their adipose tissue skatole levels above the sensory threshold. This is particularly important, as the majority of consumers are able to perceive skatole in pork, and dislike it [8]. 

Thus, using genotype, environment, age and weight alone is not a good indication of boar taint in carcasses. Even though technology is continuously improving for the identification of boar taint on slaughter lines, there remain abattoirs which cannot afford such equipment or the trained staff required to operate them, and thus still rely on basic indicators such as weight, fatness and age of the animal. According to the correlations investigated in the present manuscript, testicle weight and tissue color are better indicators of androstenone and skatole concentrations in the adipose tissue than live weight and subcutaneous backfat depth. As these compounds are affected by the degree of testicular activity, it was thus expected that indicators of change in testicular activity would be more reliable estimators of immunocastration success and of boar taint in carcasses. While these factors should be investigated on a large scale and integrated into models which may set sorting limits for successfully immunocastrated pigs, or high-risk boar taint carcasses according to the popular genotypes before they may be used in abattoirs which cannot afford higher technologies, these physiological factors may also be integrated into other precision livestock management tools to improve the power of their indirect prediction. It may also be possible to develop color cards that can be used to distinguish testicles that might be indicative of boar taint, as is typically used in beef abattoirs for meat color and fat grading of Wagyu. These correlations should also be expanded over higher concentrations of androstenone and skatole. Such information could be used as a pre-screening tool, without cutting or damaging the carcass itself, for those carcasses requiring further investigation before determination of their sale potential.

Nonetheless, the results of the present study indicate that immunocastration was successful in suppressing testicular functioning and preventing the accumulation of androstenone and skatole in the adipose tissue of male slaughter pigs (Large White × Landrace × White Duroc maternal line and PIC© 410 terminal sire line), regardless of changes in dietary protein and ractopamine hydrochloride supplementation. Thus, in comparison to alternatives, such as entire male production and surgical castration, immunocastration, together with the provision of adequate dietary protein and use of ractopamine hydrochloride, provides a welfare-friendly technique with potentially low-cost implications, when considering the cost (and variable) effectivity of anesthesia, as well as the losses associated with the processing (or rejection) of carcasses with boar taint. 

## 5. Conclusions

Immunocastration was successful in decreasing testicular functioning, resulting in 100% of the treated animals having androstenone concentrations below the defined sensory threshold, and decreased skatole concentrations in comparison to the entire males. The dietary protein levels used in this study and ractopamine hydrochloride supplementation had no influence on the accumulation of skatole in the adipose tissue, and thus may be commercially considered to support optimum growth of immunocastrated pigs. Commercially used indicators for potential boar taint in carcasses, including body mass and carcass fatness, were not reliable indicators thereof, but testicle weight and color were better correlated with boar taint compounds. Thus, by further examining the correlations of testicular activity indictors with androstenone and skatole concentrations using mass data and incorporating this information with other physiological factors, potential pre-sorting limits may be established for identifying successful immunocastration or carcasses with a higher predisposition for boar taint.

## Figures and Tables

**Table 1:** The ingredient and nutrient composition (as-is basis) of the various dietary protein finisher diets fed to immunocastrated and entire male pigs from 20 to 24 weeks of age, with or without ractopamine hydrochloride supplementation (*n* = 120).

Ingredient Composition, g/kg	Dietary Protein Level		
	Low	Medium	High
Maize	327	266	206
Wheat bran	295	210	124
Barley meal	150	150	150
Soya oil cake (470 g CP ^1^/kg)	127	213	300
Sunflower oil cake (360 g CP ^1^/kg)	50	100	150
Canola oil	25.0	27.5	30
Limestone	14.2	13.4	12.7
Salt	4.3	4.36	4.42
L-lysine HCL	2	1.9	1.8
Vitamin and mineral premix ^2^	2	2	2
Monocalcium phosphate	1.9	1.0	0
Mycotoxin binder	1	1	1
L-threonine	0.53	0.36	0.18
Phytase enzyme	0.5	0.5	0.5
DL-methionine	0.27	0.32	0.37
Choline chloride liquid	0.13	0.13	0.13
Xylanase and β-glucanase enzyme combination	0.1	0.1	0.1
Maize gluten meal (600 g CP ^1^/kg)	0	8.34	16.7

^1^ CP: crude protein; ^2^ Vitamin and Mineral premix: Vitamin A: 5489.5 IU/kg, Vitamin D: 1005.3 IU/kg, Vitamin E: 27.6 IU/kg, Vitamin K: 2.8 mg/kg, Niacin: 22.0 mg/kg, Riboflavin 4.9 mg/kg, d-Pantothenate: 16.5 mg/kg, Vitamin B12: 22.0 mcg/kg, Zinc: 100 mg/kg, Iron: 66 mg/kg, Manganese: 25 mg/kg, Copper: 10 mg/kg, Iodine: 0.33 mg/kg and Selenium: 0.25 mg/kg.

**Table 2:** The nutrient composition (as-is basis) of the various dietary protein finisher diets fed to immunocastrated and entire male pigs from 20 to 24 weeks of age, with or without ractopamine hydrochloride supplementation. All amino acids shown are provided as their digestible values.

Calculated Nutrient Composition	Dietary Protein Level		
	Low	Medium	High
NE ^1^ pig, MJ/kg	9.2	9.2	9.2
DE ^2^ pig, MJ/kg	13.29	13.56	13.83
Crude protein, g/kg	161	208	256
Crude starch, g/kg	359	315	271
Crude fiber, g/kg	60.8	64.4	68.1
Crude fat, g/kg	49.1	48.3	47.4
Ash, g/kg	59.3	65.2	71.0
Lysine, g/kg	7.50	9.79	12.1
Methionine, g/kg	2.47	3.28	4.09
TSAA ^3^, g/kg	4.74	6.11	7.48
Tryptophan, g/kg	1.59	2.12	2.66
Threonine, g/kg	4.88	6.36	7.85
Arginine, g/kg	9.24	12.7	16.1
Isoleucine, g/kg	5.08	7.09	9.09
Leucine, g/kg	10.3	13.8	17.2
Valine, g/kg	6.11	8.12	10.1
Histidine, g/kg	3.48	4.55	5.61
Calcium, g/kg	7.51	7.5	7.49
Total phosphorus, g/kg	6.86	7.14	7.42
Available phosphorus, g/kg	2.5	2.52	2.53
Sodium, g/kg	2.0	2.0	2.0
Potassium, g/kg	9.9	11.4	12.9

^1^ NE: net energy; ^2^ ME: metabolizable energy; ^3^ TSSA: total sulphur-containing amino acids.

**Table 3:** Summary of the initial live weight, the slaughter weight and backfat depth at slaughter for 96 pig carcasses, which were selected from a larger growth study (*n* =120) investigating the effects of varying dietary protein levels, with or without ractopamine hydrochloride supplementation.

Treatment		Initial Live Weight at 16 Weeks Old (kg)	Slaughter Weight at 24 Weeks Old (kg)	Hennessey Grading Probe Backfat Depth (mm)
**Castration Status**	**Entire (*n* = 48)**	57.7 ± 0.70	130.2 ± 1.18	16.9 ± 0.42
	**Immunocastrated (*n* = 48)**	57.5 ± 0.65	127.5 ± 1.21	17.4 ± 0.43
	***p***	0.727	0.134	0.444
**Ractopamine Hydrochloride Supplementation**	**0 mg/kg (*n* = 48)**	57.3 ± 0.74	127.7 ± 1.21	17.8 ± 0.43
	**10 mg/kg (*n* = 48)**	57.9 ± 0.59	129.9 ± 1.18	16.6 ± 0.42
	***p***	0.481	0.182	0.05
**Dietary Protein**	**Low (*n* = 32)**	57.2 ± 0.89	128.7 ± 1.47	17.6 ± 0.53
	**Medium (*n* = 32)**	57.9 ± 0.88	128.7 ± 1.44	17.5 ± 0.52
	**High (*n* = 32)**	57.7 ± 0.71	129.1 ± 1.47	16.5 ± 0.53
	***p***	0.821	0.979	0.305

**Table 4:** The testicle size, CIE Lab colour value and tissue morphology traits (Least Square (LS) Mean ± Standard Error of Mean (SEM)) of immunocastrated and entire male pigs.

Parameters	Castration Status		*p*
	Entire	Immunocastrated	
**Testicle size (*n* = 96)**	**(*n* = 48)**	**(*n* = 48)**	
Paired weight (g)	536 ± 14.0	282 ± 14.4	<0.001
Paired volume (mL)	1173 ± 23.1	1186 ± 23.6	0.914
Individual length (mm)	113 ± 1.48	93.6 ± 1.52	<0.001
Individual width (mm)	61.7 ± 1.36	62.7 ± 1.39	0.599
**Testicle surface colour (*n* = 32)**	**(*n* = 16)**	**(*n* = 16)**	
L*	52.6 ± 0.66	59.1 ± 0.72	<0.001
a*	9.98 ± 0.26	6.95 ± 0.42	<0.001
b*	11.0 ± 0.14	12.4 ± 0.21	<0.001
**Seminiferous tubule (*n* = 32)**	**(*n* = 16)**	**(*n* = 16)**	
Circumference (µm)	1080 ± 23.4	921 ± 21.5	<0.001
Epithelium thickness (µm)	76.1 ± 2.27	65.4 ± 2.65	0.002

**Table 5:** The concentrations (µg/g) of androstenone, skatole and indole in the adipose tissue of immunocastrated and entire male pigs, fed varying protein diets, with or without ractopamine hydrochloride for the last 28 days of finishing. Results are reported as LS Mean ± SEM.

		Androstenone	Skatole	Indole
**Castration Status**	**Entire (*n* = 48)**	0.098 ± 0.008	0.038 ± 0.004	0.009 ± 0.0015
	**Immunocastrated (*n* = 48)**	0.029 ± 0.009	0.012 ± 0.004	0.004 ± 0.0015
	***p***	<0.001	<0.001	0.116
**Ractopamine Hydrochloride Supplementation**	**0 mg/kg (*n* = 48)**	0.061 ± 0.009	0.024 ± 0.004	0.006 ± 0.0015
	**10 mg/kg (*n* = 48)**	0.067 ± 0.008	0.026 ± 0.004	0.007 ± 0.0015
	***p***	0.590	0.567	0.334
**Dietary Protein**	**Low (*n* = 32)**	0.071 ± 0.0104	0.023 ± 0.0054	0.008 ± 0.0018
	**Medium (*n* = 32)**	0.061 ± 0.0102	0.025 ± 0.0053	0.006 ± 0.0018
	**High (*n* = 32)**	0.06 ± 0.0204	0.027 ± 0.0053	0.005 ± 0.0018
	***p***	0.671	0.754	0.571
